# The relationship between gamma-band neural oscillations and language skills in youth with Autism Spectrum Disorder and their first-degree relatives

**DOI:** 10.1186/s13229-024-00598-1

**Published:** 2024-05-07

**Authors:** Vardan Arutiunian, Megha Santhosh, Emily Neuhaus, Heather Borland, Chris Tompkins, Raphael A. Bernier, Susan Y. Bookheimer, Mirella Dapretto, Abha R. Gupta, Allison Jack, Shafali Jeste, James C. McPartland, Adam Naples, John D. Van Horn, Kevin A. Pelphrey, Sara Jane Webb

**Affiliations:** 1 Center for Child Health, Behavior and Development, Seattle Children’s Research Institute, 1920 Terry Ave., Seattle, WA 98101 USA; 2 Department of Psychiatry and Behavioral Science, University of Washington, Seattle, WA USA; 3 Institute of Human Development and Disability, University of Washington, Seattle, WA USA; 4 Center for Autism Research and Treatment, Semel Institute for Neuroscience and Human Behavior, David Geffen School of Medicine, University of California Los Angeles, Los Angeles, CA USA; 5 Department of Psychiatry and Biobehavioral Sciences, University of California Los Angeles, Los Angeles, CA USA; 6 Department of Pediatrics, Yale School of Medicine, New Haven, CT USA; 7 Yale Child Study Center, Yale School of Medicine, New Haven, CT USA; 8 Department of Neuroscience, Yale School of Medicine, New Haven, CT USA; 9 Department of Psychology, George Mason University, Fairfax, VA USA; 10 Department of Neurology, Children’s Hospital of Los Angeles, Los Angeles, CA USA; 11 School of Data Science, University of Virginia, Charlottesville, VA USA; 12 Department of Neurology, School of Medicine, University of Virginia, Charlottesville, VA USA

**Keywords:** Autism Spectrum Disorder (ASD), Gamma power, Language skills, Unaffected siblings, Excitation/inhibition balance

## Abstract

**Background:**

Most children with Autism Spectrum Disorder (ASD) have co-occurring language impairments and some of these autism-specific language difficulties are also present in their non-autistic first-degree relatives. One of the possible neural mechanisms associated with variability in language functioning is alterations in cortical gamma-band oscillations, hypothesized to be related to neural excitation and inhibition balance.

**Methods:**

We used a high-density 128-channel electroencephalography (EEG) to register brain response to speech stimuli in a large sex-balanced sample of participants: 125 youth with ASD, 121 typically developing (TD) youth, and 40 unaffected siblings (US) of youth with ASD. Language skills were assessed with Clinical Evaluation of Language Fundamentals.

**Results:**

First, during speech processing, we identified significantly elevated gamma power in ASD participants compared to TD controls. Second, across all youth, higher gamma power was associated with lower language skills. Finally, the US group demonstrated an intermediate profile in both language and gamma power, with nonverbal IQ mediating the relationship between gamma power and language skills.

**Limitations:**

We only focused on one of the possible neural contributors to variability in language functioning. Also, the US group consisted of a smaller number of participants in comparison to the ASD or TD groups. Finally, due to the timing issue in EEG system we have provided only non-phase-locked analysis.

**Conclusions:**

Autistic youth showed elevated gamma power, suggesting higher excitation in the brain in response to speech stimuli and elevated gamma power was related to lower language skills. The US group showed an intermediate pattern of gamma activity, suggesting that the broader autism phenotype extends to neural profiles.

**Supplementary Information:**

The online version contains supplementary material available at 10.1186/s13229-024-00598-1.

## Background

Autism Spectrum Disorder (ASD) is a highly heritable neurodevelopmental condition associated with difficulties in social interaction/communication, the presence of stereotyped/repetitive behavior, and restricted interest or atypical response to sensor information [1]. Although language impairment is not among the core characteristics of ASD, about 75% of children with this disorder have co-occurring language difficulties [2, 3]. Language functioning is highly heterogeneous and can vary from severe impairment (e.g., nonverbal or minimally verbal ASD) to above-average language skills [4–6]. Given the variability of language skills in this population, it is most likely that there are multiple neurobiological mechanisms that are related to language impairment in ASD [7–9]. Moreover, given the well-known broader autism phenotype, some of these autism-specific language deficits may also be presented in the first-degree relatives of children with ASD [10, 11].

One of the possible neural mechanisms related to autistic behaviors is cortical gamma-band (30–150 Hz) oscillations measured with electro- and magnetoencephalography (EEG/MEG). Animal and cellular studies with optogenetic manipulations have shown that gamma oscillations are associated with the balance between neural excitation (E) and inhibition (I) and generated mostly by gamma-aminobutyric acidergic (GABAergic) interneurons, expressing calcium-binding protein parvalbumin (PV + basket cells) [12–17]. In general, aberrant gamma activity in autistic individuals has been reported in a number of studies as a potential biomarker related to both core and co-occurring conditions of ASD [18, 19]. Additionally, animal models of autism and electrophysiological studies in combination with magnetic resonance spectroscopy have suggested that E/I imbalance and the dysfunction of the GABAergic system may be a physiological mechanism contributing to expression of autistic phenotypes [20–27]. Given the close relation of gamma oscillations to clinically relevant processes such as language processing, nonverbal IQ, and social functioning [9, 28, 29], these oscillations are of particular interest in ASD research.

A number of previous studies have demonstrated atypical gamma activity in toddlers, children, youth and adults with ASD and their first-degree relatives in response to low-level auditory as well as high-level linguistic stimuli and the relationship between these brain responses and both expressive and receptive language skills [18, 30–38]. For example, with respect to low-level auditory processing, reduced gamma power and/or inter-trial phase consistency was reported in both autistic children and their non-autistic siblings/parents, using 40 Hz amplitude-modulated tones and amplitude modulated sweeps; these altered brain responses were associated with lower receptive as well as overall language skills [30, 34]. Both reduced and elevated gamma activity in autistic adults and their first-degree relatives was also reported when presenting linguistic stimuli of different complexity, such as syllables, words, and sentences [31, 32]. Resting-state or baseline gamma oscillations were also related to both expressive and overall language abilities of toddlers with idiopathic ASD and their siblings and/or parents as well as individuals with single-gene disorders with elevated autism behaviors, such as Fragile X Syndrome [9, 39, 40], and could even be an early biomarker of further language functioning in ASD [9, 39]. Summarizing, atypical neural activity at the gamma frequency band was related to language abilities of both autistic individuals and their first-degree relatives and may be a non-invasive objective measure of language functioning in the endophenotype. Remarkably, according to the previous findings, gamma activity perhaps was not associated with a specific domain of language skills (e.g., expressive vs. receptive) in relation to the specific stage of child development (toddlers vs. youth), but rather reflected a general feature of the functioning of autistic brain in relation to overall language abilities.

The goal of the present study was to investigate the relationship between gamma activity in response to speech stimuli and language skills in a large sample of sex-matched autistic youth, typically developing (TD) youth, and unaffected siblings (US) of youth with ASD. We aimed to estimate EEG spectral power at a gamma frequency band (as the E/I balance marker [13, 41]) using a language learning paradigm. The study also explored which phenotypic characteristics could explain the possible relationship between gamma power in response to speech stimuli and language skills of the US group. The strengths of the study were fourfold. First, instead of passive paradigms with low-level auditory stimuli or perception of speech samples, we used a language learning paradigm that activates broader neural networks associated with language processing in a representative sample of participants. Second, our sample consisted of a roughly equal number of male and female individuals; this is essential, as the previous studies showed that male and female individuals can have different profiles with respect to language and communication abilities [42–46]. Third, we used a standardized formal assessment to evaluate the language skills of each child. Finally, to characterize the relationship between gamma power and language skills of the US group, we used mediation modeling that allows for probing of causal relationships between variables.

## Methods

### Participants

A total of 286 native English-speaking youth aged 7 to 18 years participated in the study: 125 autistic youth (58 female, 67 male), 121 TD youth (61 female, 60 male), and 40 US of youth with ASD (24 female, 16 male); all participants from the US group were siblings of autistic youth from the present study. Sex was based on parent report of sex assigned at birth. Data were collected from four sites as a part of the GENDAAR Autism Center for Excellence network, including Seattle Children’s Research Institute, Boston Children’s Hospital, the University of California in Los Angeles, and Yale University.

The study was approved by the Yale Institutional Review Board, the UCLA Office of Human Research Protection Program, Boston Children’s Hospital Institutional Review Board, USC Office for the Protection of Research Subjects, and the University of Virginia Institutional Review Board for Health Sciences Research. All performed procedures were in accordance with the Declaration of Helsinki. All minor children provided verbal assent to participate in the study and were informed that they can withdraw from the study at any time during the experiment. A written consent form was obtained from a parent of each child participating in the study.

### Clinical and behavioral assessment

All youth with ASD were diagnosed with the Autism Diagnosis Observation Schedule—Second Edition (ADOS-2) [47] and DSM–IV–TR [48]. Participants were included in the study if they had *either* verbal or nonverbal IQ ≥ 70 based on the Differential Ability Scales—Second Edition (DAS-II) School Aged Cognitive Battery [49]. Exclusion criteria were twin status, history and/or presence of known chromosomal syndromes/single-gene conditions related to autism (e.g., Fragile X Syndrome), co-occurring neurological conditions (e.g., epilepsy), significant visual and auditory impairments, or sensory-motor difficulties that would prevent completion of study procedures. TD children had no first or second degree family members with ASD, and no elevation of autism traits according to parent report on the Social Responsiveness Scale—Second Edition (SRS-2) [50] (T-score < 60) or the Social Communication Questionnaire [51] (raw score < 11). Adaptive skills were measured with the Vineland Adaptive Behavior Scales—Second Edition (Vineland-II); standard scores in communication, socialization, and daily living skills domains were calculated for each child [52]. Language abilities were scored with the Clinical Evaluation of Language Fundamentals—Fourth Edition (CELF-4) [53], a standardized assessment tool that covers basic structural language skills at different linguistic levels (vocabulary, morphosyntax, semantics, and pragmatics) in both production and comprehension. The participants were administered only with the tests that were necessary to calculate CELF-4 Core Language Standard Score, which was used as a measure of overall language skills. All participants from the TD group as well as the US group had normal language skills based on the CELF-4 Core Language Standard Score. Demographic information is presented in Table 1.

**Table 1:** Demographic information of participants, *M (SD)*

Characteristics	Group			Statistics (*t*-tests between groups)		
	ASD (*N* = 125)	TD (*N* = 121)	US (*N* = 40)	ASD vs. TD	ASD vs. US	TD vs. US
Age (months)	147.9 (34.7)	157.7 (34.1)	140.1 (32.6)	*t* = –2.22, *p* = 0.03*	*t* = 1.3, *p* = 0.20	*t* = 2.9, *p* = 0.004**
Sex (*N* of female participants)	58	61	24	χ^2^(1) = 0.17, p = 0.68	χ^2^(1) = 1.84, p = 0.17	χ^2^(1) = 0.90, p = 0.34
SRS-2 (raw total score)	90.3 (28.7)	17.3 (22.6)	25.8 (23.6)	*t* = 21.3, *p* < 0.001***	*t* = 13.7, *p* < 0.001***	*t* = –1.9, *p* = 0.06
CELF-4 (Core Language SS)	91.86 (21.37)	110.81 (11.30)	110.07 (11.62)	*t* = –8.73,* p* < 0.001***	*t* = –6.87,* p* < 0.001***	*t* = 0.34, *p* = 0.72
Vineland-2						
Communication SS	75.7 (11.8)	99.3 (13.7)	95.0 (13.9)	*t* = –14.6,* p* < 0.001***	*t* = –7.9, *p* < 0.001***	*t* = 1.6, *p* = 0.10
Socialization SS	71.2 (11.6)	101.7 (13.0)	101.4 (14.9)	*t* = –19.3, *p* < 0.001***	*t* = –11.7, *p* < 0.001***	*t* = 0.1, *p* = 0.92
Daily living skills SS	75.7 (13.6)	97.8 (14.1)	96.5 (17.4)	*t* = –12.5,* p* < 0.001***	*t* = –6.9, *p* < 0.001***	*t* = 0.4, *p* = 0.65
Verbal IQ	99.1 (18.7)	113.5 (16.1)	112.5 (11.0)	*t* = –6.4, *p* < 0.001***	*t* = –5.5, *p* < 0.001***	*t* = 0.4, *p* = 0.66
Nonverbal IQ	99.8 (16.8)	108.6 (15.0)	111.9 (16.8)	*t* = –4.3, *p* < 0.001***	*t* = –3.9, *p* < 0.001***	*t* = –1.1, *p* = 0.27
ADOS-2						
CSS Total	6.7 (2.0)	–	–	–	–	–
CSS SA	6.8 (2.1)	–	–	–	–	–
CSS RRB	6.4 (2.9)	–	–	–	–	–

Key: SS = Standard Score; CSS = Calibrated Severity Score; SA = Social Affect; RRB = Restrictive and Repetitive Behaviors

### Experimental paradigm and procedure

**Fig. 1:**
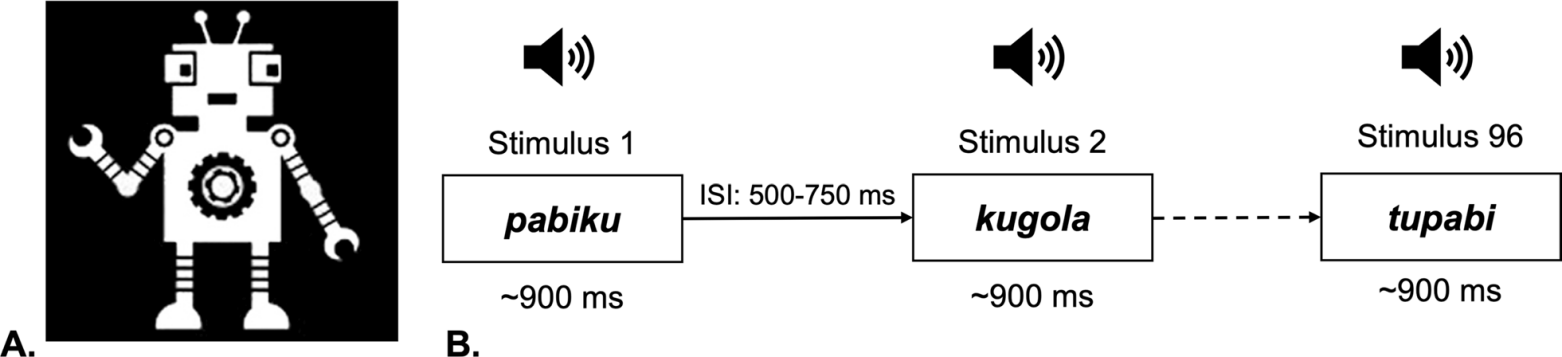
Structure of the experiment

The implicit word segmentation paradigm from [54–59] was used. During the first (exposure) phase, participants were presented auditorily with three-syllabic pseudowords generated from the set of 12 different phonemes (e.g., *pa-bi-ku*); resulting in 180 exposures over approximately 2.5 min duration. The second (test) phase consisted of 96 trials; duration was 2 min 16 s. Analyses were restricted to the second (test) phase of experiment. Each trial consisted of a tree-syllabic pseudoword with the average duration of ~ 900 ms, followed by a 500–750 ms intertrial interval. A random half of these trials (48) used the same pseudowords presented in the first phase during exposure (e.g., *pa-bi-ku*), i.e., ‘familiar’ items. The remaining random 48 trials were constructed by combining the last syllable of each familiar pseudoword with the first two phonemes of other pseudowords (e.g., *pa-bi-ku* and *go-la-tu* became *ku-go-la* and *tu-pa-bi*), i.e., ‘unfamiliar’ items. The auditory stimuli were presented using a speaker (Logitech speaker system X320) with the same loudness across all participants and sites (65 dB). Children were instructed to look at the screen and to listen carefully to the ‘robot language’. A static robot was presented on the screen simultaneously with the auditory stimuli during the experiment. Figure 1 represents a schematic structure of the experiment.

### EEG data acquisition and processing

At all four sites, EEG data were collected with EGI 128-channel Net Amps 300 system with HydroCel nets (Magstim EGI Inc., Eugene OR), using Net Station 4.4.2, 4.5.1, or 4.5.2 with a standard Net Station acquisition template.^1^ Nets were available without outriders (eye electrodes 125, 126, 127, and 128) for participants with facial sensory sensitivities. The participant’s behavior was video recorded during EEG collection. Data were collected at 500 Hz sampling rate, referenced to Cz electrode (vertex), and impedances were < 50 kΩ.

**Fig. 2:**
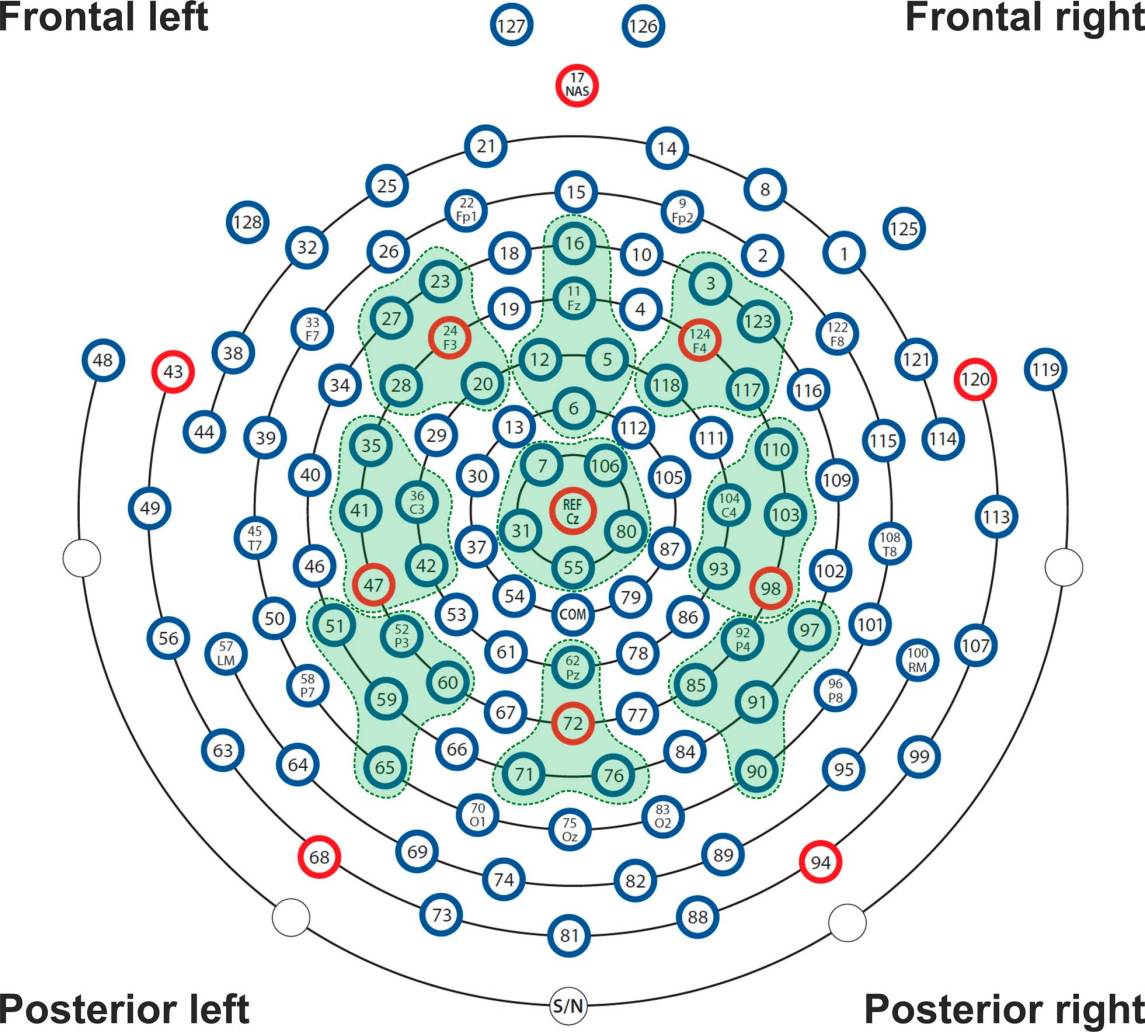
EEG montage with channels indicated. Channel numbers for regions are (1) frontal left (20, 23, 24, 27, 28), (2) frontal midline (5, 6, 11, 12, 16), (3) frontal right (3, 117, 118, 123, 124), (4) central left (35, 36, 41, 42, 47), (5) central midline (7, 31, 55, 80, 106), (6) central right (93, 98, 103, 104, 110), (7) posterior left (51, 52, 59, 60, 65), (8) posterior midline (62, 71, 72, 76), (9) posterior right (85, 90, 91, 92, 97)

To calculate power spectral density (PSD) for the gamma-band frequency range (35–54.99 Hz) we used the Batch EEG Automated Processing Platform, BEAPP [60] in MATLAB 2021a, consisting of: (1) format the MFF file for Matlab; (2) band-pass filter 1–100 Hz; (3) down sampling from 500 to 250 Hz; (4) implementation of the Harvard Automated Preprocessing Pipeline for EEG (HAPPE) module for artifact detection and rejection [61], including removal of 60 Hz line noise, rejection of bad channels, wavelet enhanced thresholding, Independent Component Analysis (ICA) with automated component rejection, bad channel interpolation, and re-referencing to average; (5) segmentation of the continuous file into 1 s epochs (each epoch consisted of one three-syllabic pseudoword); (6) rejection of bad segments (± 40 μV); (7) calculation of the PSD using Hanning window on clean segments. We focused on the low gamma frequency band (35–54.99 Hz) to avoid potential effects of 60 Hz line noise and the notch filter used for its removal. A total of nine regions of interest (ROIs) were used for the analysis as depicted in Fig. 2. PSD was calculated for each electrode, averaged within each ROI, and normalized with natural logarithm transformation.

The US group had fewer artifact-free epochs in the test ‘familiar’ condition in comparison to ASD and TD groups; ASD and TD groups did not differ in the number of artifact-free epochs in any condition: ‘familiar’ items, *ASD, M*_epoch_ = 43, range 30–47; *TD, M*_epoch_ = 43, range 34–47; *US, M*_epoch_ = 41, range 12–45; *F*(2, 283) = 5.61, *p* = 0.004. ‘Unfamiliar’ items, *ASD, M*_epoch_ = 43, range 28–47; *TD, M*_epoch_ = 43, range 33–47; *US, M*_epoch_ = 42, range 11–46; *F*(2, 283) = 1.19, *p* = 0.30.

EEG and behavioral data were available for all participants.

### Statistical analysis

All linear models used in the analysis were estimated in R [62] with the *lme4* package [63]; mediation models were estimated with the *sem* [64] and *lavaan* [65] packages. The data were plotted with *ggplot2* [66], and *semPlot* [67] packages; the figures, representing neural responses, were created with python data visualization library *matplotlib* [68]. The structure of the models will be specified further in the Results section.

## Results

### Group and condition differences in gamma power

In order to assess between-group difference in gamma power in response to speech stimuli, we fitted a linear mixed-effect model for each ROI with gamma power as a dependent variable, condition (familiar vs. unfamiliar), group (ASD, TD and US; the intercept corresponded to the ASD group), condition × group interaction, and sex as main effects, and participants as a random intercept; the structure of the model was as follows: *lmer(power* ~ *condition* × *group* + *sex* + *(1 | ID), data* = *data, control* = *lmerControl(optimizer* = *"Nelder_Mead")).* A correction for multiple comparisons (false discovery rate, FDR) was applied to the models, and *p*-values for significant predictors were corrected with *p.adjust.method* in R. Additional analysis addressing ROI as a factor in the model, as well as ROI vs. composite whole-head EEG measure (gamma power averaged across all ROIs) can be found in Additional file 1.

**Fig. 3:**
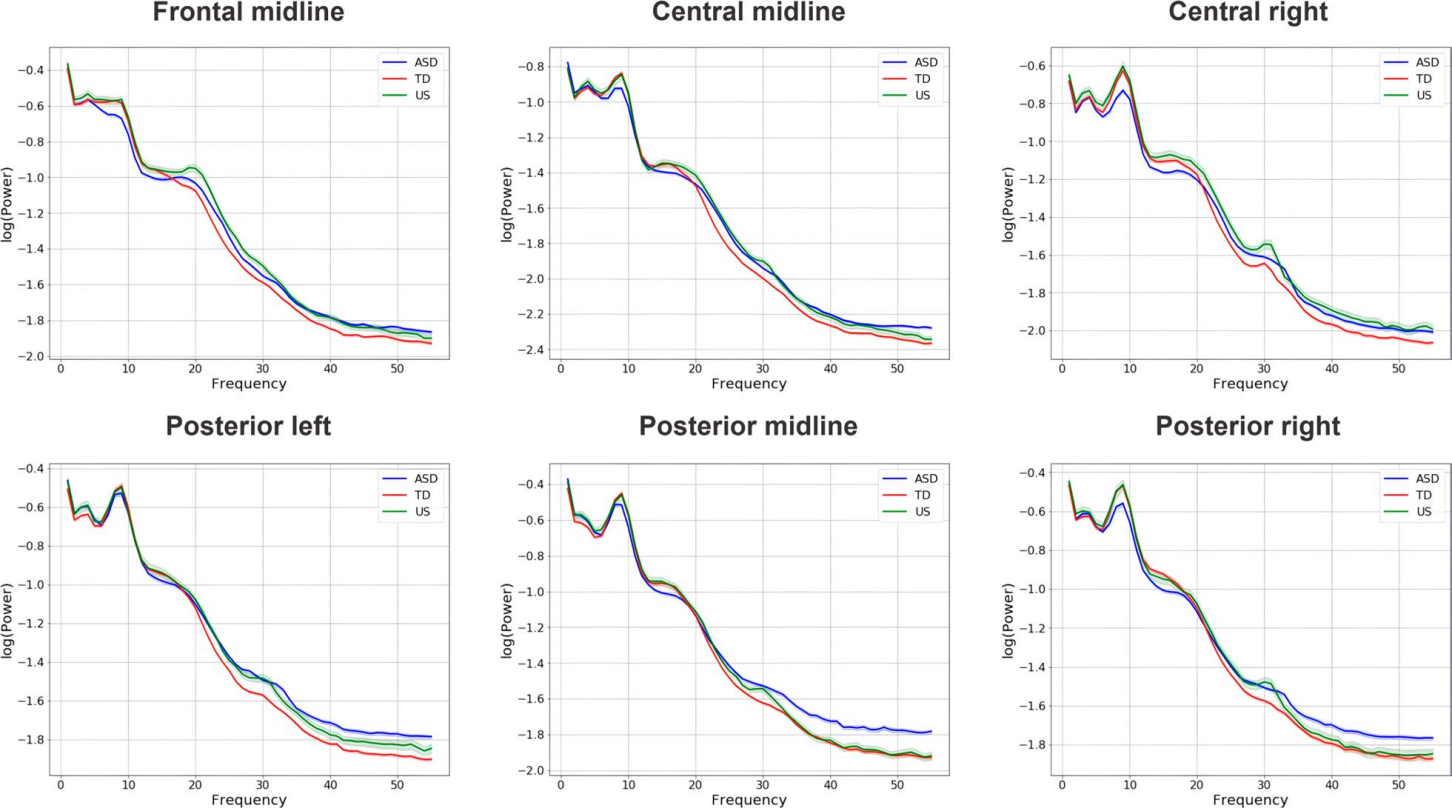
Absolute power spectra for six regions of interest which showed statistically significant between-group differences in gamma power (35–54.99 Hz) in response to speech stimuli. The plots represent the broad frequency range (ASD = Autism Spectrum Disorder; TD = typically developing; US = unaffected siblings of children with ASD)

**Fig. 4:**
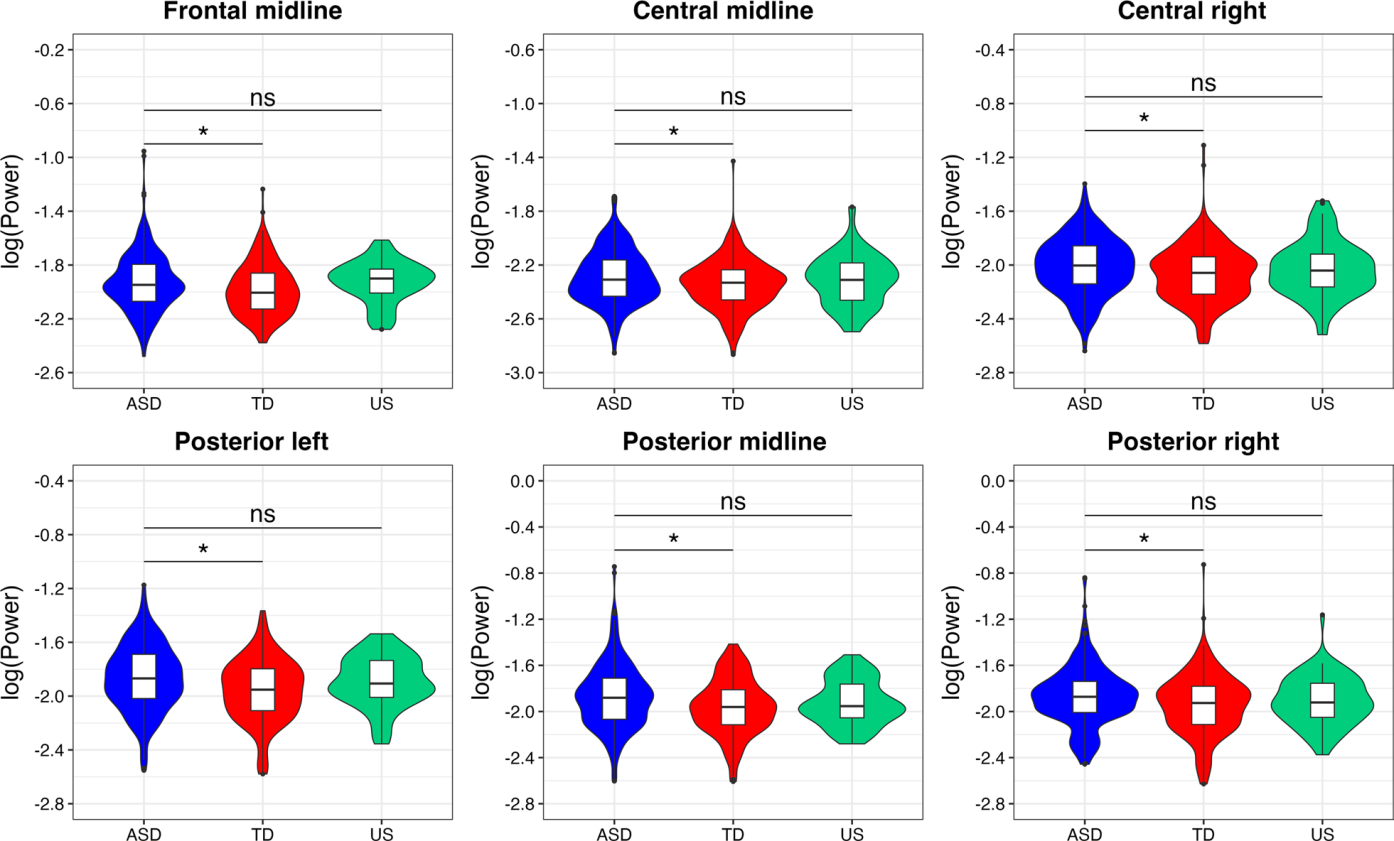
Between-group differences in gamma power (35–54.99 Hz) in response to speech stimuli in six regions of interest (ASD = Autism Spectrum Disorder; TD = typically developing; US = unaffected siblings of children with ASD). The significance is labeled with *p < 0.05, **p < 0.01, ns = non-significant. All *p*-values are FDR-corrected

The results showed neither between-condition difference in the gamma power nor condition × group interaction in any ROI, indicating that the pattern of neural response for familiar and unfamiliar pseudowords was similar in ASD, TD, and US groups (Table 2). At the same time, the results revealed a statistically significant between-group difference in gamma power in six out of nine ROIs after applying FDR-correction (Fig. 3): autistic youth had elevated power in comparison to TD youth (Fig. 4, see Table 2). No statistical difference was found between the ASD and US groups in any ROI. Descriptive statistics of mean values of gamma power in these six regions showed that the US group had lower power in comparison to autistic youth but higher power when comparing to TD youth (Table 3). A main effect of sex was identified in four ROIs, so as the power of gamma activity was higher in the male group.

**Table 2:** Between-group and between-condition differences in gamma power (35–54.99 Hz) in nine regions of interest (ASD = Autism Spectrum Disorder; TD = typically developing; US = unaffected siblings of children with ASD). All significant *p*-values are FDR-corrected

Frontal ROI	Est	SE	*t*	*p*	Central ROI	Est	SE	*t*	*p*	Posterior ROI	Est	SE	*t*	*p*
*Left*					*Left*					*Left*				
Condition	0.00	0.00	0.94	0.34	Condition	–0.00	0.00	–0.36	0.71	Condition	0.00	0.00	0.72	0.46
Group (TD)	–0.04	0.02	–1.71	0.08	Group (TD)	–0.00	0.00	–1.40	0.16	Group (TD)	–0.00	0.00	–3.06	0.01*
Group (US)	–0.03	0.03	–0.95	0.34	Group (US)	0.00	0.00	0.45	0.65	Group (US)	–0.00	0.00	–0.37	0.70
Sex	–0.01	0.02	–0.43	0.66	Sex	0.00	0.00	2.76	0.01*	Sex	0.00	0.00	3.73	< 0.01***
Condition × group (TD)	–0.00	0.00	–1.11	0.26	Condition × group (TD)	0.00	0.00	1.29	0.19	Condition × group (TD)	–0.00	0.00	–0.10	0.91
Condition × group (US)	–0.00	0.00	–0.63	0.52	Condition × group (US)	0.00	0.00	0.67	0.50	Condition × group (US)	–0.00	0.00	–0.30	0.76
*Midline*					*Midline*					*Midline*				
Condition	–0.00	0.00	–0.16	0.89	Condition	–0.00	0.00	–0.47	0.63	Condition	0.00	0.00	0.41	0.68
Group (TD)	–0.00	0.00	–2.11	0.04*	Group (TD)	–0.05	0.02	–2.24	0.04*	Group (TD)	–0.08	0.03	–2.62	0.04*
Group (US)	0.00	0.00	0.10	0.91	Group (US)	–0.01	0.03	–0.43	0.66	Group (US)	–0.04	0.04	–0.97	0.33
Sex	–0.00	0.00	–1.11	0.26	Sex	0.04	0.02	1.90	0.09	Sex	0.05	0.02	1.71	0.12
Condition × group (TD)	0.00	0.00	0.72	0.47	Condition × group (TD)	0.00	0.00	1.04	0.29	Condition × group (TD)	–0.00	0.00	–0.68	0.49
Condition × group (US)	–0.00	0.00	–0.23	0.81	Condition × group (US)	0.00	0.00	0.65	0.51	Condition × group (US)	0.00	0.00	1.51	0.13
*Right*					*Right*					*Right*				
Condition	–0.00	0.00	–0.39	0.69	Condition	0.00	0.00	0.87	0.38	Condition	0.00	0.00	0.25	0.80
Group (TD)	–0.00	0.00	–1.67	0.09	Group (TD)	–0.00	0.00	–2.16	0.04*	Group (TD)	–0.00	0.00	–2.22	0.04*
Group (US)	–0.00	0.00	–1.22	0.22	Group (US)	–0.00	0.00	–0.19	0.84	Group (US)	–0.00	0.00	–0.55	0.58
Sex	–0.00	0.00	–0.31	0.75	Sex	0.00	0.00	3.32	0.003**	Sex	0.00	0.00	4.21	< 0.01***
Condition × group (TD)	0.00	0.00	0.26	0.79	Condition × group (TD)	0.00	0.00	0.09	0.92	Condition × group (TD)	–0.00	0.00	–0.40	0.68
Condition × group (US)	0.00	0.00	0.96	0.33	Condition × group (US)	–0.00	0.00	–0.28	0.77	Condition × group (US)	0.00	0.00	0.88	0.37

The ASD group is in the Intercept, and it is used as a comparison group (ASD vs. TD; ASD vs. US)

**Table 3:** Numeric values of gamma power (log transformed) in six regions of interest for three groups of children (ASD = Autism Spectrum Disorder; TD = typically developing; US = unaffected siblings of children with ASD), *µ*V

Region of interest	Group					
	ASD (*N* = 125)		TD (*N* = 121)		US (*N* = 40)	
	M	SD	M	SD	M	SD
Frontal midline	–1.93	0.22	–1.98	0.19	–1.93	0.15
Central midline	–2.29	0.20	–2.35	0.18	–2.31	0.19
Central right	–2.01	0.21	–2.07	0.21	–2.03	0.21
Posterior left	–1.86	0.24	–1.96	0.23	–1.89	0.19
Posterior midline	–1.87	0.28	–1.96	0.24	–1.92	0.19
Posterior right	–1.87	0.26	–1.94	0.26	–1.91	0.21

### The relationship between gamma power and language skills

In order to examine whether variations in gamma power in response to speech stimuli had behavioral or clinical relevance, we fitted a linear mixed-effect model for those significant six ROIs with gamma power as a dependent variable, CELF-4 Core Language Standard Score as a factor (behavioral measure of language skills), and participants as a random intercept, according to the formula: *lmer(power* ~ *CELF-4 Core Language Standard Score* + *(1 | ID), data* = *data).*

**Fig. 5:**
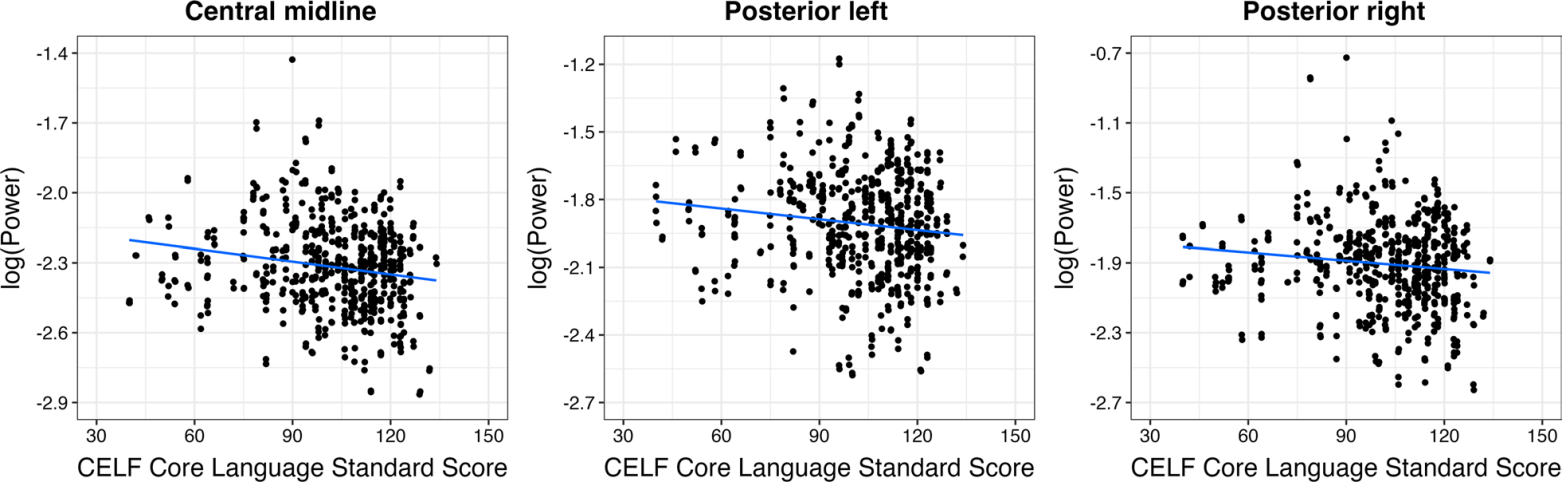
The relationship between gamma power (35–54.99 Hz) in response to speech stimuli and language skills of youth

The results revealed a significant relationship between gamma power in three out of six ROIs and behavioral language abilities: higher gamma was associated with lower language skills (Fig. 5): *central midline,* Est = –1.826e-03, SE = 5.922e-04, *t* = –3.08, *p* = 0.002; *posterior left,* Est = –1.595e-03, SE = 7.268e-04, *t* = –2.19, *p* = 0.03; *posterior right,* Est = –1.575e-03, SE = 7.814e-04, *t* = –2.02, *p* = 0.04. After correction for multiple comparisons (*p.adjust.method* in R) these effects remained significant: FDR-corrected *p*-values are 0.006, 0.04, 0.04 for central midline, posterior left, and posterior right ROIs, respectively. Other ROIs did not show statistically significant effects: *frontal midline,* Est = –1.160e-03, SE = 6.214e-04, *t* = –1.87, *p* = 0.06; *central right,* Est = –8.117e-04, SE = 6.580e-04, *t* = –1.23, *p* = 0.22; *posterior midline,* Est = –1.274e-03, SE = 7.977e-04, *t* = –1.60, *p* = 0.11.

#### Considering age and sex

As previous studies have demonstrated that the gamma power can change during child development [69–74] and we observed a main effect of sex in four ROIs, we fitted a linear mixed-effect model for three ROIs that showed significant effects (central midline, posterior left, posterior right) to assess the relationship between gamma power and language skills while accounting for age and sex: *lmer(power* ~ *CELF-4 Core Language Standard Score* + *age* + *sex* + *(1 | ID), data* = *data).* We applied a correction for multiple comparisons to the models, so all *p*-values are FDR-corrected. The full models’ outcomes are presented in Table 4. After correction for multiple comparisons and accounting for age and sex, the relationship between gamma power and language skills for central midline ROI remained significant, Est = –0.00, SE = 0.00, *t* = –2.79, *p* = 0.015. Age and sex effects were not related to gamma power (see Table 4). For the posterior left and right ROIs, the association between gamma power and language skills was not significant when controlling for age and sex.

**Table 4:** The relationship between gamma power (35–54.99 Hz) and language skills of youth when accounting for age and sex (CELF-4 Core Language Standard Score = Language SS). All significant *p*-values are FDR-corrected

Predictors	Central midline				Posterior left				Posterior right			
	Est	SE	t	*p*	Est	SE	*t*	*p*	Est	SE	*t*	*p*
(Intercept)	–2.11	0.08	–27.29	< 0.001***	–1.66	0.09	–18.06	< 0.001***	–1.74	0.10	–17.51	< 0.001***
Language SS	–0.00	0.00	–2.79	0.015*	–0.00	0.00	–1.59	0.148	–0.00	0.00	–1.45	0.148
Age	–0.00	0.00	–1.14	0.256	–0.00	0.00	–3.13	0.006**	–0.00	0.00	–1.86	0.096
Sex	0.04	0.02	1.70	0.089	0.10	0.03	3.75	< 0.001***	0.12	0.03	4.18	< 0.001***
*Random effects*												
σ^2^	0.00	0.00	0.00									
τ_00_ _ID_	0.03	0.05	0.06									
ICC	0.97	0.98	0.94									
N _ID_	286	286	286									
Observations	571	571	571									
Marginal R^2^/Conditional R^2^	0.045/0.968				0.088/0.981				0.077/0.945			

#### Summary

Between-group comparisons showed that autistic youth had elevated gamma power in comparison to TD youth. Higher gamma power was related to lower language skills in the central midline ROI.

### The phenotype of unaffected siblings of children with ASD

**Fig. 6:**
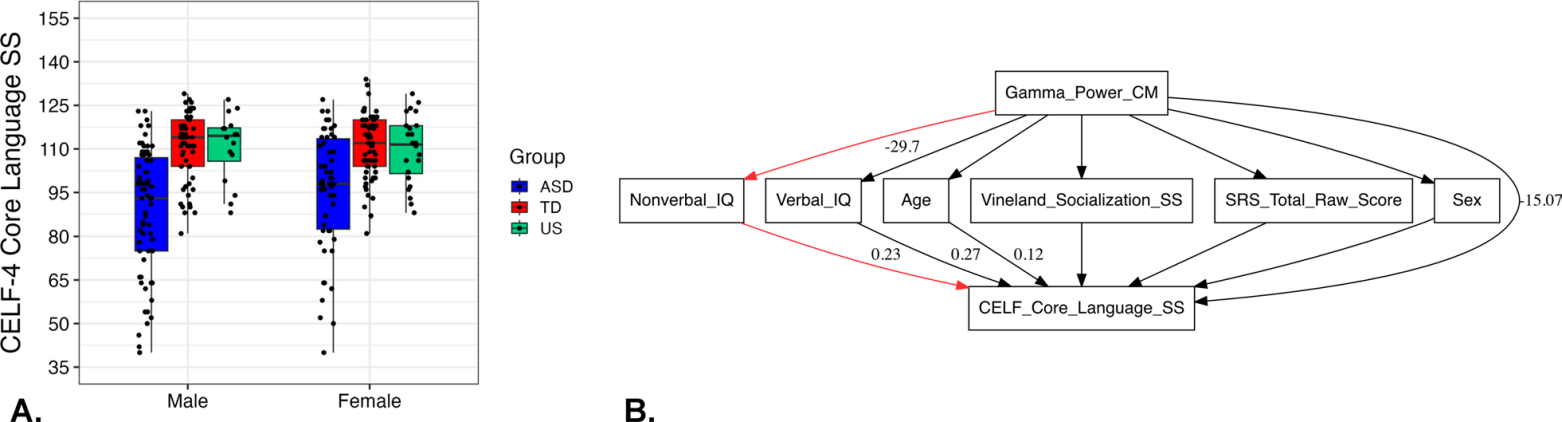
Language skills of unaffected siblings of children with Autism Spectrum Disorder: **A**—a comparison of CELF-4 Core Language Standard Score in three groups of children (ASD = Autism Spectrum Disorder; TD = typically developing; US = unaffected siblings of children with ASD); **B**—a mediation model for central midline region of interest: red path represents a statistically significant indirect relationship between gamma power and language skills via nonverbal IQ

This follow-up post-hoc analysis focused on the US group specifically, as this group demonstrated an ‘intermediate’ neural phenotype between the ASD and TD groups. To assess the language skills of the US group in comparison to ASD and TD groups, we fitted a linear model with main effects of group (the intercept corresponded to the US group), sex (as assigned at birth), and group × sex interaction; sex was included into the model as the previous studies showed that male and female individuals can have different profiles with respect to language and communication abilities [42–46]. The structure of the model was as follows: *lm(CELF-4 Core Language Standard Score* ~ *group* + *sex* + *group* × *sex, data* = *data).* The results revealed a main effect of group, indicating that the US group had significantly higher language skills in comparison to the ASD group, Est = –12.24, SE = 1.41, *t* = –8.68, *p* < 0.001; but significantly lower language skills when comparing to TD participants, Est = 6.44, SE = 1.42, *t* = 4.54, *p* < 0.001 (see Table 5, Fig. 6A). It is important to note that all participants from the US group had language skills at or above average on the CELF-4 Core Language Standard Score (*M* = 110, range 88–129).

**Table 5:** A comparison of CELF-4 Core Language Standard Scores in three groups of children (ASD = Autism Spectrum Disorder; TD = typically developing; US = unaffected siblings of children with ASD)

Predictors	CELF-4 Core language standard score			
	Estimate	SE	*t*	*p*
(Intercept)	104.37	1.13	92.75	< 0.001***
ASD group	–12.24	1.41	–8.68	< 0.001***
TD group	6.44	1.42	4.54	< 0.001***
Sex (female)	1.08	1.13	0.96	0.339
ASD group × sex (female)	2.80	1.41	1.99	0.048*
TD group × sex (female)	–1.22	1.42	–0.86	0.391
Observations	286			
R^2^/R^2^ adjusted	0.262/0.248

To explore which phenotypic characteristics inform the relationship between gamma power and language skills of US participants, we fitted a mediation model (for the ROI in which the association between gamma power and CELF-4 Core Language Standard Score remained significant when controlling for age, sex, and the correction for multiple comparisons) and included sex, age, nonverbal IQ, verbal IQ, Vineland Socialization Standard Score, and SRS-2 total raw score as mediators. The model assessed the direct effects of gamma power on language skills as well as indirect effects through all mediators included in the models. Also, the model calculated the overall indirect effect and the total effect. The full model outcome is presented in Table 6, and standardized estimates of path coefficients are depicted in Fig. 6B.

**Table 6:** The output of the mediation model for central midline region of interest

Regressions	Estimate	SE	z-value	P ( >|z|)	CI (lower)	CI (upper)
Nonverbal IQ ~ gamma power (a1)	–29.702	10.455	–2.841	0.004**	–50.193	–9.211
Verbal IQ ~ gamma power (a2)	–8.861	7.138	–1.241	0.214	–22.851	5.129
Age ~ gamma power (a3)	–17.173	20.494	–0.838	0.402	–57.339	22.994
Vineland Socialization ~ gamma power (a4)	5.889	10.037	0.587	0.557	–13.784	25.561
SRS total score ~ gamma power (a5)	–3.668	15.789	–0.232	0.816	–34.614	27.278
Sex ~ gamma power (a6)	–0.048	0.332	–0.145	0.885	–0.700	0.603
CELF-4 Core Language Standard Score ~						
Nonverbal IQ (b1)	0.232	0.057	4.055	< 0.001***	0.120	0.344
Verbal IQ (b2)	0.269	0.084	3.213	0.001**	0.105	0.433
Age (b3)	0.115	0.029	3.946	< 0.001***	0.058	0.172
Vineland Socialization (b4)	0.047	0.060	0.791	0.429	–0.070	0.164
SRS total score (b5)	0.009	0.038	0.227	0.820	–0.066	0.083
Sex (b6)	–1.741	1.799	–0.968	0.333	–5.268	1.785
Gamma power (c)	–15.071	5.505	–2.738	0.006**	–25.860	–4.282
*Variances*						
Nonverbal IQ	238.447	39.200	6.083	< 0.001***	161.615	315.278
Verbal IQ	111.153	18.274	6.083	< 0.001***	75.338	146.969
Age	916.246	150.630	6.083	< 0.001***	621.017	1211.476
Vineland Socialization	219.782	36.132	6.083	< 0.001***	148.964	290.599
SRS total score	543.863	89.410	6.083	< 0.001***	368.621	719.104
Sex	0.241	0.040	6.083	< 0.001***	0.163	0.319
CELF-4 Core Language Standard Score	57.730	9.491	6.083	< 0.001***	39.129	76.332
*Defined parameters*						
Indirect effect 1 (a1*b1)	–6.890	2.961	–2.327	0.020*	–12.693	–1.086
Indirect effect 2 (a2*b2)	–2.385	2.060	–1.158	0.247	–6.423	1.652
Indirect effect 3 (a3*b3)	–1.977	2.412	–0.820	0.412	–6.705	2.751
Indirect effect 4 (a4*b4)	0.277	0.589	0.471	0.638	–0.877	1.431
Indirect effect 5 (a5*b5)	–0.032	0.194	–0.162	0.871	–0.413	0.349
Indirect effect 6 (a6*b6)	0.084	0.585	0.143	0.886	–1.063	1.231
Overall indirect effect	–10.923	4.422	–2.470	0.014*	–19.590	–2.255
Total effect	–25.994	6.495	–4.002	< 0.001***	–38.723	–13.265

The results of the mediation model showed a statistically significant indirect effect of nonverbal IQ as a mediator between gamma power and language skills of US participants, Est = –6.89, SE = 2.96, *z* = –2.33, *p* = 0.02, C.I. [–12.69, –1.09]. Also, the model revealed significant overall indirect and total effects: overall indirect effect, Est = –10.92, SE = 4.42, *z* = –2.47, *p* = 0.01, C.I. [–19.59, –2.25]; total effect, Est = –25.99, SE = 6.49, *z* = –4.00, *p* < 0.001, C.I. [–38.72, –13.26]; SRMS = 0.180, AIC = 3828.398, TLI = –0.027, CFI = 0.450, power (1—β) = 0.499. The same mediation analysis was provided for the ASD and TD groups to assess whether the mediation role of nonverbal IQ in the relationship between gamma power and language skills is universal for all groups. The results did not reveal any indirect paths between gamma power and language skills in both groups, indicating hypothetically that this effect can be specific to the US group (see Additional file 1 with the full model outcomes for the ASD and TD groups).

As the models revealed an indirect or mediation effect of nonverbal IQ, we provided follow-up between-group comparison in nonverbal IQ, using the same structure of model as for CELF-4 Core Language Standard Score: linear model with main effect of group (the intercept corresponded to the US group), sex (as assigned at birth), and group × sex interaction. The results showed a main effect of group, indicating that the US group had a significantly higher nonverbal IQ in comparison to the ASD group, Est = –7.01, SE = 1.39, *t* = –5.04, *p* < 0.001; however, no difference was found between US and TD groups: Est = 1.83, SE = 1.39, *t* = 1.31, *p* = 0.19. No main effect of sex and an effect of group × sex interaction was identified: *sex,* Est = –0.06, SE = 1.11, *t* = –0.05, *p* = 0.96; *ASD, female,* Est = –0.70, SE = 1.39, *t* = –0.51, *p* = 0.61; *TD, female,* Est = 0.80, SE = 1.40, *t* = 0.57, *p* = 0.57.

#### Summary

US individuals had higher language skill in comparison to youth with ASD but lower in comparison to TD youth. In the US group, nonverbal IQ mediated the relationship between gamma power and language skills.

## Discussion

The present study investigated neural activity at the gamma frequency band in response to speech stimuli in autistic youth and their first-degree relatives, as well as the relationship between this neural activity and language skills. In general, results revealed an elevation in EEG spectral power at the gamma frequency band in youth with ASD and their siblings and showed that variability in gamma activity was associated with language skills measured in formal assessment. The analysis in the US group showed that nonverbal IQ mediated the relationship between brain response and language abilities.

In accordance with the previous findings [75–77], we showed altered gamma activity in youth with ASD when comparing them to TD youth but extend this to neural activity during speech processing. Given the nature of the task (perception of speech stimuli presented auditorily), we proposed that the main brain areas generated gamma activity were temporal regions in the left and right hemispheres, which is consistent with the previous studies that identified alterations in gamma response in the auditory cortex of autistic individuals [30, 32, 34, 74]. In general, gamma-band abnormalities in autism were reported in multiple studies [19, 20, 35, 78–80] and were considered as a potential biomarker [18, 19] related to both core characteristics and co-occurring conditions in ASD, including language functioning [30]. Gamma oscillations are one of the indexes of E/I balance, and they arise from the inhibition of pyramidal cells via binding the inhibitory GABAergic neurotransmitter [12–14]. Thus, increased spectral power at the gamma frequency range may reflect increased E/I ratio [25]. This, in turn, may result in a selective enhancement of excitation and increased ‘noise’ in the cortex, which in turn impacts synaptic plasticity during development and results in less effective information processing [24, 25]. Elevated excitatory activity in the autistic brain may also explain a high rate of epilepsy in this population: it is known that the rate of epilepsy in autistic individuals is approximately 20% [81] whereas in general population it is ~ 1%. The hypothesis of increased E/I ratio and altered gamma activity in ASD was supported by both animal models of autism and cellular studies of neural tissues [82–84].

The observed elevation in EEG spectral power at the gamma frequency range during a speech task in autistic youth and the intermediate pattern of gamma activity in the US participants was related to behavioral language functioning: higher gamma power was associated with lower language skills. A number of previous studies have demonstrated that gamma oscillations play a significant role in the local networks involved in language processing. For instance, in the left temporal region, which is a crucial cortical area for speech processing, gamma oscillations are specifically associated with coding temporal fine units of speech [41, 85–87] and analyzing the properties of a sound in the short temporal integration windows [88, 89]. In addition, biophysical models of neural computations suggested that gamma oscillations are sufficient for phoneme identification during speech processing [41]. Thus, all these types of speech processing could be affected by alterations in gamma activity due to an E/I imbalance. While previous findings have shown a tight relationship between gamma power and age [69–74], we revealed a significant association between neural activity at the gamma band and language in our broad developmental-range sample when accounting for age. In general, given the specificity of this neural activity the changes in gamma power during child development could be associated with the maturation of GABAergic inhibitory neurotransmission and age-related changes of E/I balance [70]. This maturation starts very early in development by switching from a depolarizing to hyperpolarizing action of GABA receptors, that is, excitatory-to-inhibitory shift of GABA receptors [90–92]. Previous findings have shown that gamma power increases rapidly during first years of life [78] with a less rapid increase in the adolescence and early adulthood [93] and then decreases in the late brain development [94]. Therefore, age-related changes of gamma activity in the early stages of brain development may reflect neural maturation that promotes efficient cognitive development, including language development.

The analysis of the US group identified an intermediate pattern of gamma activity as well as language skills based on CELF-4 Core Language Standard Score. This is consistent with the previous findings that showed either intermediate or similar to ASD pattern of brain activity in the first-degree relatives of individuals with ASD [95, 96] as well as lower language and communication skills in comparison to neurotypical population [10, 11]. The findings supported the hypothesis of broader autism phenotype [97], pointing to a highly heritable nature of language processing in autistic individuals. To understand the complexity of the relationship between gamma power and language skills in the US group, we also provided mediation modeling. The results demonstrated that EEG spectral power at the gamma frequency range in response to speech stimuli was related to language skills via nonverbal IQ. It is important to note that the US individuals from our study had both normal language skills and nonverbal IQ. Previous studies with autistic individuals have shown a relationship between nonverbal IQ and language [2] as well as nonverbal IQ and gamma power [29], but this is the first study that identified the mediation role of nonverbal IQ. These findings highlight the specific phenotypical characteristic of US individuals, contributing to better understanding of variability in functioning in this population.

## Limitations

The findings of the study should be considered within the context of several limitations. First, we want to highlight that the study focused on *one of the possible* neural mechanisms of language functioning in ASD and the broader autistic phenotype and did not include the analysis of other types of brain responses. Future studies would benefit from addressing the neural activity at all frequency bands and regional specificity of these patterns. This could contribute to understanding of the complex neural system underlying language variability in families with child with autism. Second, the US group consisted of smaller number of participants in comparison to ASD or TD groups (US = 40 youth, ASD = 125 youth, TD = 121 youth); thus, given the low statistical power of the mediation analysis the replication is needed. Finally, due to the timing issue in EEG system we have provided only non-phase-locked analysis. It is important to note, however, that we have adjusted the timing window as much as it was possible during the post-acquisition processing to address the error as it manifested in the systems we used. Following the disclosure of the timing error by EGI we incorporated StimTracker event markers, which use auditory and photodiode sensors and 5v inputs to the digital inputs of the amplifiers to assess ground-truth timing accuracy. These analyses revealed (1) that the timing drift was not present at all sites; (2) in those recording sessions where there was drift, it was no larger than 25 ms across a 45 min recording session. However, as we cannot confirm the specific timing error for each individual nor the stability of the delay across the whole of the recording, we adjusted our analysis window such that we are able to expect that 90% of the 1 s trial reflects brain activity during the perception of the pseudoword. Thus, future studies using the same EEG system should address this issue and check the timing drift for providing as much precise analysis as possible.

## Conclusions

To conclude, the study demonstrated, *first,* an elevated EEG spectral power at the gamma frequency range in response to speech stimuli in autistic youth and the intermediate pattern of activity in unaffected siblings (US) of youth with ASD. These results may support the hypothesis of E/I imbalance in autistic individuals. However, there can be alternative interpretations of our results, such as, for example, more involvement of attention of autistic individuals in the task and increased gamma power due to increased attention. *Second,* the findings revealed that elevated gamma power was related to lower language skills. *Finally,* the phenotypic analysis of the US group showed that the link between gamma activity and language skills was mediated by nonverbal IQ.

### Supplementary Information

**Additional file 1**. Supplementary results.

## Abbreviations

ASD: Autism Spectrum Disorder
EEG: Electroencephalography
MEG: Magnetoencephalography
GABA/GABAergic: Gamma-aminobutyric acidergic
PV: Parvalbumin
TD: Typically developing
US: Unaffected siblings
ADOS-2: Autism diagnosis observation schedule-second edition
DAS-II: Differential ability scales-second edition
SRS-2: Social responsiveness scale-second edition
Vineland-II: Vineland adaptive behavior scales-second edition
CELF-4: Clinical evaluation of language fundamentals-fourth edition
PSD: Power spectral density
BEAPP: Batch EEG automated processing platform
HAPPE: Harvard automated preprocessing pipeline for EEG
ICA: Independent component analysis
ROI(s): Region(s) of interest

## Footnotes

1 An advisory notice related to timing offsets for the GES 300 systems with firewire cameras was released in 2016. Due to the potential impact on some of our data acquired, we do not provide stimulus locked results. https://www.egi.com/knowledge-center/item/63-ges-300-and-firewire-cameras

## Declarations

### Availability of data and materials

The behavioral and EEG data from the current study are available via the National Institute of Mental Health Data Archive Data Collection #2021.
