# Supplementary material for: The relationship between gamma-band neural oscillations and language skills in youth with Autism Spectrum Disorder and their first-degree relatives
Source: Mol Autism. 2024 May 7;15:19. doi: 10.1186/s13229-024-00598-1 (PMC11075235; doi:10.1186/s13229-024-00598-1)
Supplement: Supplementary file 1 — Additional file 1. Supplementary results. [file 13229_2024_598_MOESM1_ESM.pdf]

Additional file 1. Supplementary results

1. Region of interest (ROI) in the model as a factor

Main results of the study were based on ROI-by-ROI analysis, so as separate models were fitted for each ROI. This supplementary analysis addresses a model that includes ROI as a factor. The structure of the model is as follows: gamma power as a dependent variable, condition, group, and condition*group interaction as main effects, sex and ROI as factors, and participants as random intercept. The results of the model are presented in Table S1.

Table S1. *Between-group difference in gamma power, considering ROI as a factor*

	Gamma power			
Predictors	***Estimate***	***Standard error***	***t***	***p***
(Intercept)	-2.03	0.02	-100.15	**<0.001**
Condition	0.00	0.00	0.20	0.843
Group_TD	-0.06	0.02	-2.60	**0.009**
Group_US	-0.02	0.03	-0.59	0.558
sex	0.05	0.02	2.18	**0.029**
ROI1	-0.28	0.01	-36.45	**<0.001**
ROI2	-0.00	0.01	-0.30	0.761
ROI3	0.20	0.01	26.12	**<0.001**
ROI4	0.08	0.01	10.73	**<0.001**
ROI5	0.19	0.01	24.22	**<0.001**
ROI6	0.13	0.01	16.46	**<0.001**
ROI7	0.12	0.01	15.25	**<0.001**
ROI8	0.13	0.01	16.52	**<0.001**
Condition*Group_TD	0.00	0.00	0.05	0.959
Condition*Group_US	0.00	0.01	0.38	0.703
**Random Effects**				
σ^2	0.02			
$\tau_{00\ SiteID}$	0.03			
ICC	0.64			
N_{SiteID}	286			
Observations	5139			
Marginal R^2 / Conditional R^2	0.301 / 0.751			

The model revealed a main effect of group in gamma power, so as the TD group had lower power when comparing to the ASD group. We also observed main effects of ROIs, pointing to a regional-specific differences in gamma power.

2. Composite whole-head EEG measure (gamma power averaged across all ROIs)

The previous model demonstrated that there is a main effect of ROI, however, there is a main effect of group as well (see Table S1). The main effect of group across all ROIs brough up the question whether the composite whole-head EEG measure (gamma power averaged across all ROIs) can be used to replicate the results of ROI-by-ROI analysis (between-group difference in gamma power as well as the relationship between gamma power and language skills). The models with the same structure as for the main analysis were used with 1) gamma power as a dependent variable, condition, group, and condition*group interaction as main effects, sex as a factor, and participants as a random intercept (to assess between-group difference); and 2) gamma power as a dependent variable, CELF Core Language SS as a predictor, sex and age as factors, and participants as a random intercept. See results in Tables S2 and S3 with models outcomes.

Table S2. *The output of the model (between-group difference in composite EEG power)*

	Gamma power			
Predictors	***Estimate***	***Standard error***	***t***	***p***
(Intercept)	-1.97	0.02	-100.38	**<0.001**
Condition	0.00	0.00	0.31	0.755
Group_TD	-0.06	0.02	-2.60	**0.010**
Group_US	-0.02	0.03	-0.59	0.558
sex	0.05	0.02	2.18	**0.029**
Condition*Group_TD	0.00	0.00	0.08	0.933
Condition*Group_US	0.00	0.00	0.60	0.548
**Random Effects**				
σ^2	0.00			
$\tau_{00\ SiteID}$	0.03			
ICC	0.98			
N_{SiteID}	286			
Observations	571			
Marginal R^2 / Conditional R^2	0.040 / 0.977			

Table S3. *The output of the model (relationship between composite EEG gamma power and language skills)*

	Gamma power			
Predictors	***Estimate***	***Standard error***	***t***	***p***
(Intercept)	-1.78	0.07	-24.43	**<0.001**
CELF Core Language SS	-0.00	0.00	-1.59	0.113
sex	0.05	0.02	2.20	**0.028**
age	-0.00	0.00	-2.78	**0.006**
**Random Effects**				
σ^2	0.00			
$\tau_{00\ \text{SiteID}}$	0.03			
ICC	0.98			
N_{SiteID}	286			
Observations	571			
Marginal R^2 / Conditional R^2	0.053 / 0.977			

For the first model, we revealed similar effects as for ROIs analysis: main effect of group, so that the TD group had lower power, and a main effect of sex, as the male group had higher power. At the same time, for the second model, we did not reveal a relationship between gamma power averaged across all ROIs and language skills, pointing to the idea that this effect can be a regional-specific.

3. Mediation models for the ASD and TD groups

The main analysis showed that the group of unaffected siblings (US) of youth with ASD showed an intermediate pattern (between the ASD and TD groups) in both behavioral language skills (CELF-4 Core Language Standard Score) and neural functioning (lower gamma power in comparison to the ASD group but higher when comparing to TD group). To explore which phenotypic characteristics inform the relationship between gamma power and language skills of US participants, we provided mediation analysis. The model assessed the direct effects of gamma power on language skills as well as indirect effects through all mediators included in the models (age, sex, nonverbal IQ, verbal IQ, Vineland Socialization Standard Score, and SRS-2 total raw score). The results showed that nonverbal IQ played a mediation role in the relationship between gamma power and language skills.

To assess if this indirect effect is unique or universal, we provided the same mediation models for the ASD and TD groups. The results did not show any indirect paths between gamma power and language skills in both groups, indicating hypothetically that this effect can be specific to the US group. See Tables S4 and S5 with full model outcomes.

Table S4. The output of the mediation model for central midline region of interest (TD group).

**Regressions**	**Estimate**	**SE**	**z-value**	**P (>|z|)**	**CI (lower)**	**CI (upper)**
Nonverbal IQ ~ gamma power (a1)	–5.912	5.692	–1.039	0.299	–17.069	–5.244
Verbal IQ ~ gamma power (a2)	–0.927	6.262	–0.148	0.822	–13.200	11.347
Age ~ gamma power (a3)	–15.389	13.515	–1.139	0.255	–41.877	11.099
Vineland Socialization ~ gamma power (a4)	–2.911	4.953	–0.588	0.557	–12.618	6.797
SRS total score ~ gamma power (a5)	–23.718	8.576	–2.766	**0.006****	–40.526	–6.910
Sex ~ gamma power (a6)	–0.532	0.190	–2.798	**0.005****	–0.905	–0.159
CELF-4 Core Language Standard Score ~						
Nonverbal IQ (b1)	0.138	0.036	3.808	**<0.001*****	0.067	0.210
Verbal IQ (b2)	0.331	0.033	10.019	**<0.001*****	0.266	0.396
Age (b3)	0.024	0.015	1.586	0.113	–0.006	0.054
Vineland Socialization (b4)	0.067	0.042	1.594	0.111	–0.015	0.148
SRS total score (b5)	–0.013	0.024	–0.540	0.589	–0.060	0.034
Sex (b6)	0.234	1.088	0.215	0.830	–1.899	2.367
Gamma power (c)	–9.397	3.201	–2.936	**0.003****	–15.671	–3.124
**Variances:**						
Nonverbal IQ	216.193	20.566	10.512	**<0.001*****	175.883	256.502
Verbal IQ	261.667	24.892	10.512	**<0.001*****	212.879	310.456
Age	1218.700	115.935	10.512	**<0.001*****	991.471	1445.929
Vineland Socialization	163.696	15.572	10.512	**<0.001*****	133.174	194.217
SRS total score	490.735	46.684	10.512	**<0.001*****	399.237	582.234
Sex	0.241	0.023	10.512	**<0.001*****	0.196	0.286
CELF-4 Core Language Standard Score	63.154	6.008	10.512	**<0.001*****	51.379	74.929
**Defined parameters:**						
Indirect effect 1 (a1*b1)	–0.819	0.817	–1.002	0.316	–2.420	0.782
Indirect effect 2 (a2*b2)	–0.307	2.074	–0.148	0.882	–4.371	3.757
Indirect effect 3 (a3*b3)	–0.374	0.404	–0.925	0.355	–1.166	0.418
Indirect effect 4 (a4*b4)	–0.194	0.352	–0.551	0.581	–0.883	0.495
Indirect effect 5 (a5*b5)	0.309	0.583	0.530	0.596	–0.834	1.452
Indirect effect 6 (a6*b6)	–0.125	0.581	–0.214	0.830	–1.263	1.014
Overall indirect effect	–1.509	2.435	–0.619	0.536	–6.282	3.265
Total effect	–10.906	3.823	–2.853	**0.004****	–18.399	–3.413

Fit indexes of the mediation model: SRMR = 0.137, AIC = 11516.923, TLI = 0.056, CFI = 0.494

Table S5. The output of the mediation model for central midline region of interest (ASD group).

**Regressions**	**Estimate**	**SE**	**z-value**	**P (>|z|)**	**CI (lower)**	**CI (upper)**
Nonverbal IQ ~ gamma power (a1)	–6.390	5.426	–1.178	0.239	–17.024	4.244
Verbal IQ ~ gamma power (a2)	–1.569	6.117	–0.256	0.798	–13.557	10.420
Age ~ gamma power (a3)	–20.181	10.672	–1.891	0.059	–41.098	0.736
Vineland Socialization ~ gamma power (a4)	6.558	3.661	1.792	0.073	–0.616	13.733
SRS total score ~ gamma power (a5)	11.502	9.131	1.260	0.208	–6.393	29.398
Sex ~ gamma power (a6)	–0.184	0.161	–1.142	0.254	–0.499	0.132
CELF-4 Core Language Standard Score ~						
Nonverbal IQ (b1)	0.180	0.046	3.891	**<0.001*** **	0.090	0.271
Verbal IQ (b2)	0.800	0.041	19.450	**<0.001*** **	0.719	0.880
Age (b3)	0.019	0.024	0.786	0.432	–0.028	0.065
Vineland Socialization (b4)	0.161	0.069	2.341	**0.019***	0.026	0.296
SRS total score (b5)	–0.011	0.028	–0.387	0.699	–0.065	0.043
Sex (b6)	6.344	1.564	4.056	**<0.001*** **	3.278	9.410
Gamma power (c)	–1.244	3.874	–0.321	0.748	–8.836	6.348
**Variances:**						
Nonverbal IQ	281.840	26.513	10.630	**<0.001*** **	229.875	333.805
Verbal IQ	358.193	33.696	10.630	**<0.001*** **	292.150	424.236
Age	1090.413	102.577	10.630	**<0.001*** **	889.365	1291.461
Vineland Socialization	128.284	12.068	10.630	**<0.001*** **	104.631	151.937
SRS total score	798.144	75.083	10.630	**<0.001*** **	650.984	945.304
Sex	0.248	0.023	10.630	**<0.001*** **	0.202	0.293
CELF-4 Core Language Standard Score	136.910	12.879	10.630	**<0.001*** **	111.667	162.153
**Defined parameters:**						
Indirect effect 1 (a1*b1)	–1.153	1.023	–1.127	0.260	–3.157	0.852
Indirect effect 2 (a2*b2)	–1.255	4.893	–0.256	0.798	–10.845	8.335
Indirect effect 3 (a3*b3)	–0.374	0.515	–0.726	0.468	–1.384	0.636
Indirect effect 4 (a4*b4)	1.055	0.742	1.423	0.155	–0.398	2.509
Indirect effect 5 (a5*b5)	–0.123	0.331	–0.370	0.711	–0.772	0.527
Indirect effect 6 (a6*b6)	–1.165	1.060	–1.099	0.272	–3.242	0.913
Overall indirect effect	–3.014	5.200	–0.580	0.562	–13.205	7.177
Total effect	–4.258	6.374	–0.668	0.504	–16.751	8.236

Fit indexes of the mediation model: SRMR = 0.151, AIC = 12117.853, TLI = 0.336, CFI = 0.644.
